# The role of the microbiota-gut-brain axis and artificial intelligence in cognitive health of pediatric obstructive sleep apnea: A narrative review

**DOI:** 10.1097/MD.0000000000040900

**Published:** 2024-12-13

**Authors:** Yunjiao Lv, Yongtao Xian, Xinye Lei, Siqi Xie, Biyun Zhang

**Affiliations:** a Department of First Clinical College, Guangzhou Medical University, Guangzhou, China; b Department of Pediatrics, The First Affiliated Hospital of Guangzhou Medical University, Guangzhou, China.

**Keywords:** artificial intelligence, cognitive function, gut microbiota, machine learning, microbiota-gut-brain axis, pediatric obstructive sleep apnea

## Abstract

Pediatric obstructive sleep apnea (OSA) is a prevalent sleep-related breathing disorder associated with significant neurocognitive and behavioral impairments. Recent studies have highlighted the role of gut microbiota and the microbiota-gut-brain axis (MGBA) in influencing cognitive health in children with OSA. This narrative review aims to summarize current knowledge on the relationship between gut microbiota, MGBA, and cognitive function in pediatric OSA. It also explores the potential of artificial intelligence and machine learning in advancing this field and identifying novel therapeutic strategies. Pediatric OSA is associated with gut dysbiosis, reduced microbial diversity, and metabolic disruptions. MGBA mechanisms, such as endocrine, immune, and neural pathways, link gut microbiota to cognitive outcomes. Artificial intelligence and machine learning methodologies offer promising tools to uncover microbial markers and mechanisms associated with cognitive deficits in OSA. Future research should focus on validating these findings through clinical trials and developing personalized therapeutic approaches targeting the gut microbiota.

## 1. Introduction

Pediatric obstructive sleep apnea (OSA) is a widely prevalent sleep-related breathing disorder characterized by intermittent upper airway obstruction during sleep, leading to intermittent hypoxia (IH) and sleep fragmentation (SF). These pathophysiological disturbances result in a spectrum of serious sequelae, including neurocognitive impairment, behavioral issues, stunted growth, hypertension, cardiac dysfunction, and systemic inflammation.^[1,2]^ According to guidelines from the American Academy of Pediatrics published in 2012, the prevalence of OSA in children ranges from 1.2% to 5.7%.^[3]^ A systematic review assessing the prevalence of OSA in preschool-aged children over the past decade indicates an upward trend, with rates increasing from less than 10% to a range of 10% to 20%.^[4]^ Pediatric OSA has thus emerged as a significant public health issue, warranting heightened awareness among parents and the broader community. Substantial evidence indicates that children with OSA are at increased risk for cognitive and behavioral deficits, and this risk correlates linearly with the severity of sleep-disordered breathing.^[5]^ Early identification, the development of novel therapeutic approaches, and strategies to prevent and mitigate OSA-associated cognitive deficits in children have become critical areas of research focus.

The human microbiota constitutes a complex, diverse, and prosperous community, predominantly inhabiting the gastrointestinal tract, comprising bacteria, fungi, viruses, and archaea.^[6]^ Various factors, including genetics, epigenetics, mode of delivery, diet, lifestyle, circadian rhythms, health status, and the surrounding environment, modulate this microbiota. Through both anabolic and catabolic processes, as well as direct host-microbe interactions and signaling pathways, the microbiota significantly influences a range of physiological functions, including immunity, neurobiology, and metabolism.^[7]^ Prior studies have indicated that in individuals with OSA, whether adolescents or adults, the gut microbiome undergoes substantial alterations.^[8,9]^ A robust association exists between OSA and gut dysbiosis. The gut microbiota, often termed the “second brain,” has the potential to influence brain homeostasis under both physiological and pathological conditions through the microbiota-gut-brain axis (MGBA).^[10]^ In healthy individuals, a stable gut microbiota composition positively regulates brain function via the MGBA, pivotal in maintaining gut barrier integrity and balancing inflammation.^[11]^ Under pathological conditions, the gut microbiota can influence brain function and behavior by producing metabolic byproducts, such as short-chain fatty acids (SCFAs). Evidence suggests that gut dysbiosis is critically involved in the cognitive impairments associated with sleep disorders.^[12]^ In summary, gut microbiota imbalance may be a crucial link between OSA and cognitive dysfunction. Nevertheless, the underlying mechanisms warrant further investigation.

In recent years, the field of microbiota research has been significantly impacted by the integration of modern technologies, particularly artificial intelligence (AI) and machine learning (ML). Machine learning approaches in microbiome research have highlighted challenges such as preprocessing, feature selection, predictive modeling, and model interpretation, emphasizing the importance of these technologies in extracting valuable biological information from microbiome data.^[13]^ These advanced methodologies facilitate the analysis of complex microbiome data, enabling the identification of critical microbial species and metabolic products associated with OSA and cognitive function. For instance, support vector machines (SVM) and random forest algorithms are employed to analyze high-throughput sequencing data, uncovering critical microbiota components linked to OSA-related cognitive impairments. By combining 16S rRNA gene sequencing and machine learning, researchers have been able to establish correlations between gut microbiota and conditions like substance use disorder, shedding light on the mechanisms underlying drug addiction.^[14]^ Convolutional neural networks (CNNs) are utilized to interpret microbiota images or genomic data, providing deep insights into microbial patterns. Recurrent neural networks (RNNs) and long short-term memory (LSTM) models predict dynamic changes in the gut microbiota of OSA patients and their impact on cognitive health. Dimensionality reduction techniques such as principal component analysis (PCA) and autoencoders are applied to distill complex microbiota data, revealing underlying patterns and features. The strategic use of AI in the digital era has been extensively reviewed, emphasizing its importance in various fields, including biology and biochemistry.^[15]^ The application of AI in gastrointestinal diseases has been recognized as a critical area for improving disease diagnosis and prognosis, with machine learning playing a crucial role in analyzing microbiome data to extract relevant information.^[16]^ These AI-driven approaches significantly enhance our understanding of the microbiota-gut-brain axis in pediatric OSA and pave the way for novel therapeutic interventions.

Given the increasing prevalence and profound impact of pediatric OSA on cognitive and behavioral health, there is a pressing need to explore innovative and effective therapeutic strategies. Integrating advanced microbiota research with the robust analytical capabilities of artificial intelligence and machine learning presents promising opportunities to elucidate the complex interactions between gut microbiota and cognitive function in OSA. This review endeavors to comprehensively synthesize current knowledge regarding the microbiota-gut-brain axis and its relevance to pediatric OSA, particularly emphasizing the underlying mechanisms and the application of AI and machine learning in this domain. Furthermore, this article will examine the potential for developing novel therapeutic approaches, including the modulation of gut microbiota through prebiotics and probiotics, to ameliorate cognitive deficits associated with OSA in children. By advancing our understanding of these intricate relationships, this review aims to underscore the potential of gut microbiota alterations as novel diagnostic markers and therapeutic targets for cognitive dysfunction related to OSA.

## 2. Interaction between OSA and gut dysbiosis

The mechanisms underlying the interaction between gut dysbiosis and OSA are multifaceted. IH and SF induced by OSA may directly or indirectly perturb the gut microenvironment, resulting in alterations to the gut microbiota composition. Conversely, gut dysbiosis may exacerbate systemic health via inflammatory responses and compromised gut barrier function, which can further impair sleep quality. Moreover, the gut microbiota produces metabolic byproducts, such as SCFAs, that influence brain function. It may provide feedback on the progression of OSA via the microbiota-gut-brain (MGB) axis. These interrelated mechanisms form a complex network that collectively impacts the health status of patients with OSA.

### 2.1. Impact of OSA on gut microbiota composition

The impact of OSA on gut microbiota composition is primarily manifested as dysbiosis, which may be associated with the pathophysiological features of OSA, such as IH and SF. Current research on the gut microbiota in pediatric OSA is limited, with most experimental data from animal models. For instance, a study by Moreno-Indias et al. (2015)^[17]^ using an IH mouse model to simulate OSA found that the gut microbiota in the IH group exhibited greater diversity compared to the normoxic group, with a significant increase in obligate anaerobes after 6 weeks of IH exposure. Furthermore, the study reported that the bacterial imbalance and low-grade endotoxemia induced by IH were not reversible even 6 weeks after normal breathing was resumed, with a notable reduction in the abundance of Bacteroidetes and Proteobacteria in the IH group. Another study in China examined gut microbiota changes under varying degrees of IH exposure. The findings indicated that rats exposed to IH exhibited higher levels of Clostridiales, Lachnospiraceae, and Ruminococcaceae, as well as lower levels of Lactobacillus compared to the control group.^[18]^ Sleep fragmentation (SF), another critical feature of OSA, has also been shown to alter gut microbiota composition in animal studies. Comprehensive research by Poroyko et al. (2016)^[19]^ demonstrated that adult male C57BL/6 mice subjected to SF for 4 weeks experienced a reversible reduction in Lactobacillaceae and an increase in Lachnospiraceae and Ruminococcaceae, providing further evidence of the impact of SF on gut microbiota.

Subsequent research by Triplett et al. (2020)^[20]^ found similar alterations in Lactobacillaceae and Ruminococcaceae in SF-exposed rats, although changes in Lachnospiraceae depended on the specific intestinal region sampled. In another study, Maki et al. (2020)^[21]^ analyzed fecal samples from SF-exposed rats and observed that bacteria producing SCFAs exhibited varying abundances at different stages of sleep disruption. These results suggest that SF has a significant impact on SCFA-producing bacteria. Clinical studies have also supported these findings. Notably, Wu et al (2022)^[22]^ investigated the gut microbiota of children aged 4 to 6 years with OSA and reported a reduction in SCFA-producing bacteria and an increase in pathogenic bacteria. This is consistent with findings from a study on the gut microbiota in adults with OSA (2019).^[23]^

### 2.2. Interaction between gut microbiota and OSA

The gut microbiota is widely recognized for its pivotal role in regulating metabolism, immunity, circadian rhythms, and other biological processes. Notably, alterations in the gut microbiota can potentially influence sleep parameters or patterns. A series of animal studies have underscored this, particularly in the context of OSA. For instance, a study on fecal microbiota transfer (FMT) in a mouse model provided compelling evidence. It revealed that changes in gut microbiota diversity could impair central respiratory response, thereby exacerbating IH symptoms, a key feature of OSA.^[24]^ Similarly, Badran et al. (2020)^[25]^ transplanted fecal matter from C57BL/6J mice exposed to either IH or room air into normal C57BL/6J mice. After 3 weeks of FMT treatment, significant differences were observed in the gut microbiota composition between the FMT-IH and FMT-room air groups, with FMT-IH mice exhibiting prolonged sleep duration and increased sleep cycle frequency. These findings strongly suggest that alterations in gut microbiota may indeed play a significant role in the pathogenesis of OSA.

In an innovative approach to unraveling the complex relationship between the gut microbiota, its related metabolites, and OSA, Yan et al. (2023)^[26]^conducted a genome-wide association study on OSA using data from the Finnish Gene Project. They also integrated gut microbiota data from the largest genome-wide association study database on gut microbiota and screened metabolites related to the gut microbiota from the Human Metabolome Database (HMDB). They made a groundbreaking discovery through a bidirectional Mendelian Randomization (MR) study and multivariable MR analysis (MVMR) on 196 gut microbiota species and 83 microbiota-derived metabolites. They found that *Ruminococcaceae* (ucG009) and the genus_*Subdoligranulum* were positively correlated with an increased risk of OSA. Conversely, the genera *Ruminococcaceae*, *Coprococcus*2, _*Eggerthella*, and_*Eubacterium* (*xylanophilum*_group) were negatively correlated with OSA risk. Among the 83 assessed metabolites, 3-*dehydrocarnitine*, *dehydroepiandrosterone* sulfate, and leucine were identified as potential independent risk factors for OSA. Reverse MR analysis indicated suggestive associations between OSA exposure and 6 microbiota taxa. This MR analysis provided independent evidence for the causal relationship between gut microbiota, their derived metabolites, and OSA, offering a novel perspective on their genetic-level connections. These findings imply that OSA prevention may be guided by timely modulation of the gut microbiota and emphasize the importance of screening for OSA in patients with gut microbiota dysbiosis.

## 3. Mechanisms of the microbiota-gut-brain axis (MGBA)

The MGBA constitutes a sophisticated bidirectional communication network between the gut microbiota and the central nervous system (CNS), orchestrating the interactions between the CNS and the enteric nervous system. The MGBA has garnered increasing recognition as a critical physiological system in maintaining overall health and modulating the functions of the brain and CNS. Contemporary research has elucidated 3 primary pathways through which the MGBA mediates its effects: endocrine and metabolic, immune, and neuronal pathways.^[27]^

### 3.1. Vagal pathways in the microbiota-gut-brain axis

The vagus nerve (VN), a significant component of the parasympathetic nervous system, is a mixed nerve composed of 80% afferent and 20% efferent fibers.^[28]^ Originating from the brainstem, the VN serves as a primary regulator, innervating the gut and the enteric nervous system to facilitate bidirectional communication between the brain and the gut, thereby modulating gut function.^[29]^ Afferent VN fibers can detect and respond to metabolic and immune activities through microbial neurotransmitters, hormones, fatty acids, and cytokines. Physiological and potentially harmful products in the gut can cross the intestinal and blood-brain barriers to reach the brain. This provides another route for gut-to-brain communication and influences sleep-wake cycles.^[30]^

Administration of Lactobacillus johnsonii has been shown to improve gastric vagus nerve activity, which can positively impact various physiological functions. The VN plays a crucial role in modulating different bodily systems through its connections with the CNS and various organs.^[31]^ Studies have shown that vagus nerve stimulation (VNS) can lead to selective circuit modulation and reinforcement of cholinergic pathways, which are essential for enhancing nerve activity.^[32]^ Additionally, transcutaneous auricular VNS has been demonstrated to effectively improve motor function post-stroke, indicating the potential of noninvasive methods to influence nerve function.^[33]^ Furthermore, VNS has been linked to neuroprotection and improved outcomes in conditions such as acute ischemic cerebral injury, highlighting its role in promoting nerve health and function.^[34]^

Moreover, the use of VNS has shown promise in enhancing memory and cognitive function, suggesting a broader impact on neurological processes beyond motor function.^[35]^ VNS has also been associated with modulating sympathetic-vagal balance, which can influence pain intensity and autonomic functions in conditions like fibromyalgia.^[36]^ The application of VNS has been explored in various contexts, including stroke rehabilitation, obesity management, and even as a potential therapeutic approach in COVID-19-related acute respiratory distress syndrome.^[37–39]^

Ingestion of *Lactobacillus rhamnosus* was demonstrated to regulate the expression of gamma-aminobutyric acid (GABA) receptors in the brain through a vagus nerve-dependent pathway, thereby alleviating depression and anxiety.^[40]^ GABAergic neurons are key in regulating non-rapid eye movement and rapid eye movement sleep. Benzodiazepines, such as zolpidem, target GABA receptors and are used to treat insomnia.^[41]^ Lactobacillus reuteri has been found to enhance synaptic plasticity in mouse models of autism spectrum disorder via oxytocinergic and dopaminergic signaling in a VN-dependent manner.^[42]^ However, the specific mechanisms by which certain bacteria communicate with the brain via the VN necessitate further investigation.

Neurotransmitters produced in the gut also have a signaling role in the brain through the VN. For instance, 5-hydroxytryptamine (5-HT) receptors on vagal fibers may facilitate specific pathways in the CNS. Selective serotonin reuptake inhibitors exert antidepressant effects by stimulating the VN, which relies on the activity of vagal fibers.^[43]^ Chronic VNS has been shown to induce changes in neurotransmitter levels, such as GABA, 5-HT, and norepinephrine, further affecting sleep.^[44]^ Inflammatory signals from the gut can also be transmitted to the brain via the VN. Rao et al. (2017)^[45]^ demonstrated that the gut microbiota could ameliorate anorexia by inhibiting IL-1β-mediated signaling in the hypothalamus through the VN. These findings collectively suggest that a variety of signals from the gut impact sleep and cognition through the vagal pathway.

Conversely, chronic intermittent hypoxia and SF can lead to decreased levels of neurotransmitters that promote sleep and cognition, such as GABA and serotonin, resulting in an imbalance in the typical gut ecosystem. This ultimately leads to gut dysbiosis and neuronal inflammation in the brain.^[46]^

### 3.2. Immunological pathways in the microbiota-gut-brain axis

The gastrointestinal tract, the largest immune organ in the human body, plays a critical role in the MGBA. Evidence increasingly suggests that the immune system is integral to MGBA signaling. Studies involving germ-free mice and mice treated with broad-spectrum antibiotics have demonstrated the close relationship between gut microbiota and intestinal immunity.^[47]^ The gut microbiota interacts with the immune system through various mechanisms, including the local habitat, microbe-derived metabolites (such as SCFAs, secondary bile acids, and amino acid metabolites), and other bioactive molecules such as microbe-associated molecular patterns.^[48]^ These interactions regulate local gut immunity, influence the CNS via systemic circulation, and mediate effects through microglia, which are crucial in neurodevelopmental, neuropsychiatric, and neurodegenerative diseases.^[49]^

The gut microbiota modulates the permeability of both the blood-brain barrier (BBB) and the intestinal barrier, preventing harmful substances in the gut lumen from entering the bloodstream.^[50]^ Dysbiosis may disrupt the gut barrier, permitting the translocation of microbes or microbial components like *lipopolysaccharide* (LPS) into the circulation. The immune system’s recognition of such immunogenic endotoxins triggers systemic inflammation.^[51]^ Elevated levels of pro-inflammatory molecules in the circulation can compromise the integrity of the BBB, diminishing its ability to exclude LPS and inflammatory cytokines. LPS entering the brain binds to Toll-like receptor 4 on microglia, producing pro-inflammatory cytokines and potentially damaging hippocampal neurons in the inflammatory milieu.^[52]^ Khalifa et al. (2019)^[53]^ have shown that plasma extracellular vesicles can disrupt the BBB in children with OSA, thereby contributing to cognitive impairment. Moreover, recent murine studies indicate that fecal microbiota transplantation can correct age-related immune dysfunction, while similar transplants from older to younger mice negatively affect CNS functions. These findings not only underscore the importance of the microbiota-gut-brain axis in aging but also propose the potential role of a “youthful” microbiota in preserving or enhancing cognitive function in later life, a revelation that could significantly impact our understanding of cognitive health in the elderly.^[54]^

In summary, the gut microbiota, in its direct regulation of intestinal and systemic immune homeostasis, significantly influences neuroinflammation and potentially contributes to the low-grade systemic inflammation associated with neuropsychiatric disorders. This direct control over immune homeostasis by the gut microbiota is a crucial risk factor for neurocognitive impairment. Additionally, the gut microbiota’s modulation of the function of CNS-resident immune cells profoundly impacts neurodevelopment and neuroinflammation, further highlighting its power and influence on health and disease.^[55]^

### 3.3. Endocrine and metabolic pathways in the microbiota-gut-brain axis

#### 3.3.1. Hypothalamic-pituitary-adrenal axis

The hypothalamic-pituitary-adrenal (HPA) axis, a critical endocrine pathway, is an integral part of the MGBA. It is central to the biological response to stress and plays a pivotal role in sleep regulation. Research indicates that male germ-free mice exhibit a hyper-reactive HPA axis in response to stress, underscoring the microbiota’s significant role in HPA axis regulation. Chronic elevation of cortisol levels can disrupt the gut microbiota composition and increase gastrointestinal permeability, thereby negatively impacting gut microbiota function. This dysbiosis and bacterial translocation, a process where bacteria move from the gut to other parts of the body, result in chronic systemic inflammation, which subsequently activates the HPA axis, leading to a cascade of health issues. Moreover, the HPA axis interacts with the VN. Studies in rodents have shown that VN stimulation enhances the expression of corticotropin-releasing hormone mRNA in the hypothalamus.^[56]^ Stress and HPA axis activity can also influence gut microbiota composition.^[57]^

#### 3.3.2. Neurotransmitters and metabolic products

Neuroregulators that promote the wakefulness cycle include acetylcholine (ACh), 5-HT, dopamine, norepinephrine, hypocretin, and histamine, which exert excitatory effects on the tuberomammillary nucleus.^[58]^ Conversely, GABA, melatonin, adenosine, and glutamate are amino acids that facilitate sleep by acting on receptors located in the ventrolateral preoptic nucleus. Among these neuroregulators, 5-HT, ACh, norepinephrine, dopamine, melatonin (MT), and GABA are also produced and metabolized by the gut microbiota, thereby directly or indirectly stimulating afferent gut neurons and interfacing with the CNS.^[59]^

GABA, an amino acid integral to sleep promotion, also contributes to the prevention of anxiety, stress, and emotional imbalance. Studies on germ-free animals have indicated that the microbiota can modulate circulating GABA levels. Experimental evidence has shown that specific strains of Lactobacillus and Bifidobacterium (*Lactobacillus* reuteri PBS072 and *Bifidobacterium* breve BB077) can elevate GABA levels and enhance cognitive functions such as short-term memory, attention, and executive function.^[60]^ Additional research is necessary to validate the effects of GABA-producing bacteria on sleep enhancement and treating sleep disorders.

5-HT is a neurotransmitter for regulating mood, sleep, appetite, learning, and memory. It is synthesized by enterochromaffin cells in the gut, with more than 90% of the body’s 5-HT originating from the gastrointestinal tract. Bacteria from families such as *Clostridiaceae* and *Turicibacteraceae* are capable of secreting 5-HT. Recent studies have demonstrated that *Lactobacillus* rhamnosus significantly increases 5-HT levels in the prefrontal cortex and hippocampus of rats exposed to chronic, unpredictable mild stress.^[61]^ Research by Yao et al^[62]^ showed that Ganoderma lucidum promotes sleep and elevates hypothalamic 5-HT levels in mice.

MT, a hormone pivotal in regulating the sleep-wake cycle, operates through its receptors, MT1 and MT2, which belong to the G-protein coupled receptor family. MT is secreted by the pineal gland and the gut, skin, bone marrow, and other organs.^[63]^ Gao et al^[64]^ found that sleep deprivation disrupts the microbiota composition and reduces plasma MT levels, while MT supplementation in sleep-deprived mice ameliorates jejunal dysbiosis. Further investigation by Wang et al^[52]^ revealed that gut microbiota metabolism mediates the neuroprotective effects of MT on cognitive impairments induced by sleep deprivation in mice. They identified a potential mechanism involving the downregulation of *Lachnospiraceae* NK4A136 and butyrate in the colon, alongside the inflammatory impact of *Aeromonadaceae* and LPS colonization in sleep deprivation mice. The relationship between gut-derived and pineal-derived MT and their respective roles in regulating the sleep-wake cycle warrants further exploration.

SCFAs, including butyrate, propionate, and acetate, are microbial metabolites derived from the gut microbiota’s fermentation of complex plant polysaccharides. Recent studies have explored the role of SCFAs in gut-brain communication and their potential to support BBB integrity.^[65]^ SCFAs have been implicated in sleep regulation; for instance, higher fecal propionate levels are associated with more extended periods of uninterrupted sleep in infants.^[66]^ Additionally, Wang et al^[67]^ found an inverse relationship between propionate concentrations and wakefulness after sleep onset in children. The influence of propionate on sleep across different age groups requires further elucidation. Butyrate has been linked to memory, cognition, mood, and metabolism.^[65]^ Tributyrin, a butyrate prodrug, has been shown to enhance non-rapid eye movement sleep in rats via vagal pathways in the portal vein region.^[68]^ A recent study in young adults revealed a positive correlation between sleep quality and the relative abundance of butyrate-producing taxa.^[69]^ These findings underscore the critical role of the gut microbiota in SCFA metabolism and its potential impact on sleep regulation.

In conclusion, these interactions illustrate how the gut microbiota influences neural, immune, metabolic, and endocrine signals through the MGBA, thereby affecting cognitive function. This understanding lays the groundwork for developing novel therapeutic strategies to ameliorate OSA-related cognitive impairments by modulating the balance of the gut microbiota.

## 4. Applications of modern technologies in gut microbiota research

Advancements in modern technology have significantly transformed the field of gut microbiota research, providing researchers with unprecedented insights into the intricate interactions between the gut microbiome and host health. Technologies such as high-throughput sequencing, bioinformatics, and various ML and AI algorithms have become essential tools in unraveling the complexities of the gut microbiota and its dynamic relationship with the host.^[70,71]^

These cutting-edge technologies enable researchers to conduct comprehensive analyses of the gut microbiota composition, functional potential, and the dynamic interplay between the microbiome and host physiology.^[72]^ By integrating multi-omic data, such as metagenomics, metatranscriptomics, and metabolomics, researchers can gain a holistic understanding of the gut microbiome and its impact on human health.^[70]^ Machine learning approaches have been instrumental in constructing predictive models for various aspects of gut microbiota research, including predicting individual microbiota status, response to interventions, disease risk, and more.^[73]^ Moreover, the application of machine learning in gut microbiota studies has the potential to revolutionize personalized medicine by identifying predictive microbiota biomarkers and developing tailored therapeutic strategies, particularly in conditions like type 1 diabetes and nonalcoholic fatty liver disease.^[74]^ Machine learning algorithms can leverage expanding datasets generated by high-throughput technologies to enhance their predictive power and applicability in understanding the gut microbiome. Furthermore, machine learning-based analyses have shed light on the intricate relationships between the gut microbiota and various health conditions, including cardiovascular disease, colorectal cancer, chronic inflammatory diseases, and even behavioral disorders like autism spectrum disorder and substance abuse.^[75]^These studies highlight the critical role of machine learning in deciphering the complex interactions between the gut microbiota and human health.

### 4.1. Gut microbiota data analysis

AI and ML algorithms have significantly enhanced the ability to analyze complex gut microbiota data. Among these, SVM and random forest (RF) algorithms are particularly valuable. SVM, a supervised learning model, excels in classification and regression tasks by identifying the optimal hyperplane that separates data points from different classes. This method has been effectively used to identify key microbial species and metabolites linked to specific health conditions. For instance, studies have utilized SVM to distinguish gut microbiota profiles of patients with OSA from those of healthy individuals, uncovering distinct microbial markers associated with disease severity.^[14]^

The use of RF algorithms has been instrumental in pinpointing specific microbial components associated with improved cognitive function in patients with OSA.^[76]^ RF, as an ensemble learning method, has been applied to uncover microbial taxa and metabolic pathways that are predictive of cognitive outcomes in OSA patients, allowing for the identification of microbial components linked to enhanced cognitive function.^[77]^ This highlights the significance of RF in elucidating the intricate relationship between the gut microbiota and cognitive health in individuals with OSA. These algorithms facilitate the identification of critical microbial components, providing insights into the gut-brain axis and its implications for cognitive health.

### 4.2. Pattern recognition and prediction

Pattern recognition and prediction are critical for understanding the dynamic changes in gut microbiota and their impact on health. CNNs and RNNs, including LSTM networks, have emerged as powerful tools for pattern recognition and prediction in understanding the dynamic changes in gut microbiota and their impact on health. CNNs, originally designed for image recognition tasks, have been adapted to analyze microbiome data by converting genomic sequences and microbial abundance profiles into image-like representations. This innovative approach has enabled researchers to identify intricate patterns within microbiome data associated with conditions such as OSA and cognitive functions.^[78]^

On the other hand, recurrent neural networks, including LSTM networks, have shown promise in capturing temporal dependencies in microbiome data, allowing for the prediction of future states based on past information. These networks excel in processing sequential data and have been applied in various fields for time-series analysis, making them valuable for studying the dynamic changes in gut microbiota over time.^[79]^

The utilization of CNNs and RNNs in gut microbiota research represents a significant advancement in understanding the complex interactions between microbial communities and host health. By leveraging the capabilities of these neural network architectures, researchers can uncover hidden patterns, make predictions, and gain deeper insights into the role of the gut microbiota in health and disease.

### 4.3. Data dimensionality reduction and feature extraction

Data dimensionality reduction and feature extraction are essential for managing the complexity of gut microbiota datasets. PCA and autoencoders are valuable techniques in the field of microbiome studies, particularly in distinguishing healthy and diseased states based on microbial species and metabolic pathways. PCA is utilized to reduce the dimensionality of data by transforming it into a set of linearly uncorrelated variables known as principal components, which capture the most variance within the data.^[80]^ The contribution of PCA, in addition to microbial identification, has advanced applications in distinguishing foodborne pathogens using MALDI-TOF-MS.^[81]^

Autoencoders, a type of artificial neural network, are instrumental in performing non-linear dimensionality reduction by learning efficient codings of input data. These networks excel at capturing complex, non-linear relationships within microbiota data, making them ideal for feature extraction. Studies utilizing autoencoders have successfully identified critical microbial features that are associated with conditions such as OSA and cognitive impairments, providing deeper insights into the microbiota-gut-brain axis.^[82,83]^ Autoencoders have been widely employed in various fields, including medicine and computer science, for tasks such as unsupervised clustering of radiologic and nuclear medicine image data, feature extraction in major depressive disorder risk prediction, and as explainable clustering approaches for resting-state EEG analysis.^[84]^ These applications showcase the versatility and effectiveness of autoencoders in extracting meaningful information from complex datasets.

Furthermore, autoencoders have been shown to be capable of extracting more features from spectral data compared to traditional methods, highlighting their potential in enhancing feature extraction processes. Additionally, the use of autoencoders in industrial processes, such as in robust soft sensor neural network approaches, demonstrates their ability to improve data robustness and accuracy.

Integrating AI and ML in gut microbiota research is not just a theoretical concept but a practical tool that has opened new avenues for understanding the intricate relationships between the gut microbiome, OSA, and cognitive function. Through advanced data analysis techniques such as SVM and RF, pattern recognition methods like CNNs and RNNs, and dimensionality reduction tools such as PCA and autoencoders, researchers can decode complex microbiota data to uncover critical insights. These technological advancements not only enhance our comprehension of the microbiota-gut-brain axis but also pave the way for developing novel therapeutic strategies to improve cognitive health in OSA patients.

## 5. Microbiota-targeted interventions

The primary treatment for pediatric OSA, in the absence of surgical contraindications, is adenotonsillectomy. Additional recommendations, based on clinical evaluation, include intranasal corticosteroids, montelukast, oral appliances, and weight reduction. Emerging evidence suggests that targeting gut dysbiosis could serve as a potential adjunctive therapy to standard OSA treatments, potentially reducing the associated morbidity. Current microbiota-targeted interventions encompass dietary modifications, prebiotics and probiotics, FMT, and SCFAs. These therapeutic strategies aim to enhance gut health, thereby alleviating OSA symptoms and mitigating cognitive impairment. While preclinical studies provide a theoretical foundation, systematic clinical trials are necessary to ascertain the efficacy of these interventions. Additionally, standardized and personalized treatment protocols are essential to maximize therapeutic outcomes.

### 5.1. Dietary modifications

Dietary modifications are crucial in restoring gut microbiota balance, as diet significantly influences the composition and function of the gut microbiota. Specific dietary interventions have been shown to promote the growth of beneficial microbial communities, with diets high in fiber being particularly beneficial. For example, fiber-rich diets can enhance the production of SCFAs by gut bacteria, leading to improved gut barrier function and reduced systemic inflammation.^[85]^

Studies have indicated that dietary interventions can positively modulate the gut microbiota and potentially alleviate symptoms of OSA.^[86]^ However, establishing the efficacy of specific dietary modifications in managing OSA requires robust clinical evidence. Research has shown that diet is a critical determinant of gut microbiota diversity, with profound effects on the composition of bacterial flora.^[87]^

Moreover, the gut microbiota is influenced by a combination of factors, including diet, host genetics, and sex effects.^[88]^ External factors such as diet have been recognized as significant environmental factors driving the composition and metabolism of the colonic microbiota.^[89]^ Recent studies have also highlighted the impact of sugars, salt intake, and specific dietary components on gut microbiota composition, emphasizing the intricate relationship between diet and the gut microbiota.^[90]^

In conclusion, dietary modifications represent a foundational strategy for promoting gut microbiota balance and overall health. By understanding the influence of diet on the gut microbiota, tailored dietary interventions can be developed to support microbial diversity and function, potentially offering therapeutic benefits for conditions like OSA.

### 5.2. Prebiotics and probiotics

Prebiotics and probiotics offer direct approaches to modulating the gut microbiota. Prebiotics are non-digestible food components that selectively stimulate the growth and activity of beneficial gut bacteria, while probiotics are live microorganisms that confer health benefits when administered in adequate amounts to the host. The combination of prebiotics and probiotics, known as synbiotics, may exert synergistic effects.^[91]^ Studies have shown that specific prebiotics and probiotics can modulate gut microbiota composition, reduce systemic inflammation, and enhance sleep and cognitive functions in OSA models.^[92]^ However, translating these findings into clinical practice necessitates rigorous human trials to determine the optimal strains, dosages, and treatment protocols.^[93]^

Prebiotics and probiotics have been recognized for their positive roles in promoting human nutrition and health. Prebiotics enhance the growth of beneficial bacteria, while probiotics confer various health benefits.^[94]^ The use of synbiotics, which combine prebiotics and probiotics, has been investigated in mental health studies, demonstrating potential benefits in adult mental health.^[95]^

Moreover, the influence of prebiotics and probiotics on the gut-brain axis has been explored, with these components being referred to as “psychobiotics” due to their impact on the microbiota-brain relationship.^[96]^ Additionally, the synergistic effects of synbiotics have been highlighted in promoting human health by selectively stimulating the growth of probiotics or activating bacterial metabolic pathways that benefit the host.^[97]^

In conclusion, prebiotics, probiotics, and synbiotics play crucial roles in modulating the gut microbiota and promoting overall health. While preclinical studies have shown promising results in conditions like OSA, further research through human trials is essential to establish the efficacy and optimal use of these interventions in clinical settings.

### 5.3. Fecal microbiota transplantation (FMT)

FMT is a procedure that involves transferring stool from a healthy donor to the gastrointestinal tract of a recipient with the aim of restoring a healthy gut microbiota composition. While animal studies have shown that FMT can rectify gut dysbiosis, reduce inflammation, and improve cognitive outcomes,^[98]^ its application in treating OSA is still in the early stages. Therefore, well-designed clinical trials are essential to evaluate the safety, efficacy, and long-term effects of FMT in managing OSA. Critical factors influencing the success of FMT interventions include donor selection, engraftment competition, and the microbial diversity and composition of donor feces.^[99]^

In summary, FMT shows promise as a therapeutic approach for restoring gut microbiota balance and potentially alleviating symptoms of OSA. However, further research, particularly well-controlled clinical trials, is necessary to establish the optimal protocols and outcomes of FMT in OSA management.

### 5.4. Short-chain fatty acids (SCFAs)

SCFAs, including butyrate, propionate, and acetate, are metabolites produced by the fermentation of dietary fibers by gut bacteria.^[100]^ SCFAs play crucial roles in maintaining gut health, modulating immune responses, and influencing brain function. They have been shown to support blood-brain barrier integrity and exert anti-inflammatory effects, which could be beneficial in the context of OSA.^[101]^ Research suggests that enhancing SCFA production through dietary interventions or direct supplementation may improve sleep quality and cognitive function in OSA.^[54]^ However, further studies are needed to identify the most effective strategies for increasing SCFA levels in OSA patients.

### 5.5. Optimization strategies for personalized treatment

Personalized treatment strategies are vital for maximizing the efficacy of microbiota-targeted interventions. Machine learning models, such as random forest algorithms, can be employed to optimize the combination of prebiotics and probiotics tailored to individual patients.^[102]^ These models can analyze complex microbiota data to identify the most effective microbial strains and dosages for restoring gut balance and alleviating OSA symptoms. Personalized approaches ensure that interventions are adapted to the unique microbiota composition and health status of each patient, thereby enhancing treatment outcomes.

Microbiota-targeted interventions hold significant promise as adjunctive therapies for OSA, with the potential to improve gut health and mitigate cognitive impairments associated with the condition.^[103]^ As discussed in previous sections, microbiota-targeted interventions hold promise as adjunctive therapies for OSA. However, their clinical efficacy varies significantly among individuals. Leveraging machine learning models, such as random forest algorithms, can optimize treatment strategies by analyzing complex microbiota data and identifying patient-specific microbial markers. Future research should focus on integrating these advanced analytical tools into clinical practice to create standardized yet adaptable treatment protocols. By bridging microbiota research and artificial intelligence, personalized therapies have the potential to transform OSA management.

## 6. Conclusion

Advancements in metagenomics, metatranscriptomics, and metabolomics have significantly deepened our understanding of the human microbiome and its intricate interactions with the host. While research on the gut microbiota and pediatric neurological disorders has predominantly centered on conditions such as autism spectrum disorder, epilepsy, attention-deficit/hyperactivity disorder, and adolescent depression, studies examining its role in cognitive impairments associated with OSA remain sparse. This review consolidates current research findings, elucidating the alterations in gut microbiota induced by OSA and exploring the pathological mechanisms through which the MGBA impacts brain function and cognition.

The gut microbiota exerts regulatory effects on sleep-wake behavior through the modulation of bacterial metabolites, endocrine signaling, neuronal pathways, and immune responses. Experimental studies have investigated innovative approaches to OSA treatment by targeting the gut microbiota. Preliminary findings from preclinical studies indicate that interventions such as prebiotics, probiotics, FMT, and dietary modifications yield beneficial effects in OSA animal models. Nevertheless, rigorous randomized controlled trials and further mechanistic investigations are imperative to substantiate these interventions as adjunctive therapies for OSA, potentially introducing novel therapeutic strategies in clinical practice.

## Acknowledgments

We would like to thank Peihang XU from the University of Hong Kong for assisting with the preparation and English revision of this manuscript.

## Author contributions

**Data curation:** Yunjiao Lv.

**Formal analysis:** Xinye Lei.

**Methodology:** Yongtao Xian, Xinye Lei.

**Project administration:** Biyun Zhang.

**Resources:** Yongtao Xian.

**Software:** Siqi Xie.

**Validation:** Siqi Xie.

**Writing – original draft:** Yunjiao Lv.

**Writing – review & editing:** Biyun Zhang.
